# Automatic reorientation to generate short-axis myocardial PET images

**DOI:** 10.1186/s40658-024-00673-9

**Published:** 2024-08-02

**Authors:** Yuling Yang, Fanghu Wang, Xu Han, Hui Xu, Yangmei Zhang, Weiping Xu, Shuxia Wang, Lijun Lu

**Affiliations:** 1 School of Biomedical Engineering, Southern Medical University, 1023 Shatai Road, Guangzhou, 510515 China; 2 Guangdong Provincial Key Laboratory of Medical Image Processing, Southern Medical University, 1023 Shatai Road, Guangzhou, 510515 China; 3 PET Center, Department of Nuclear Medicine, Guangdong Provincial People’s Hospital, Guangdong Academy of Medical Sciences, Southern Medical University, 106 Zhongshan Er Road, Guangzhou, 510080 China; 4 The Cancer Hospital of the University of Chinese Academy of Sciences (Zhejiang Cancer Hospital), Hangzhou, Zhejiang China; 5 Pazhou Lab, Guangzhou, 510515 China

**Keywords:** Positron emission tomography (PET), Automatic reorientation, Regional division, Image segmentation, Fitting algorithm

## Abstract

**Background:**

Accurately redirecting reconstructed Positron emission tomography (PET) images into short-axis (SA) images shows great significance for subsequent clinical diagnosis. We developed a system for automatic redirection and quantitative analysis of myocardial PET images.

**Methods:**

A total of 128 patients were enrolled for 18 F-FDG PET/CT myocardial metabolic images (MMIs), including 3 image classifications: without defects, with defects, and excess uptake. The automatic reorientation system includes five modules: regional division, myocardial segmentation, ellipsoid fitting, image rotation and quantitative analysis. First, the left ventricular geometry-based canny edge detection (LVG-CED) was developed and compared with the other 5 common region segmentation algorithms, the optimized partitioning was determined based on partition success rate. Then, 9 myocardial segmentation methods and 4 ellipsoid fitting methods were combined to derive 36 cross combinations for diagnostic performance in terms of Pearson correlation coefficient (PCC), Kendall correlation coefficient (KCC), Spearman correlation coefficient (SCC), and determination coefficient. Finally, the deflection angles were computed by ellipsoid fitting and the SA images were derived by affine transformation. Furthermore, the polar maps were used for quantitative analysis of SA images, and the redirection effects of 3 different image classifications were analyzed using correlation coefficients.

**Results:**

On the dataset, LVG-CED outperformed other methods in the regional division module with a 100% success rate. In 36 cross combinations, PSO-FCM and LLS-SVD performed the best in terms of correlation coefficient. The linear results indicate that our algorithm (LVG-CED, PSO-FCM, and LLS-SVD) has good consistency with the reference manual method. In quantitative analysis, the similarities between our method and the reference manual method were higher than 96% at 17 segments. Moreover, our method demonstrated excellent performance in all 3 image classifications.

**Conclusion:**

Our algorithm system could realize accurate automatic reorientation and quantitative analysis of PET MMIs, which is also effective for images suffering from interference.

**Supplementary Information:**

The online version contains supplementary material available at 10.1186/s40658-024-00673-9.

## Introduction

Positron emission tomography (PET) is increasingly being used routinely in the diagnosis of coronary artery disease [1, 2]. Compared to single-photon emission computed tomography (SPECT), PET has high resolution images and quantitative parameters in myocardial images [3, 4]. The myocardial PET image is a longitudinal image perpendicular to the patient’s long axis but not perpendicular to the left ventricular long axis. However, the direction of the long axis of the left ventricle is inconsistent with that of the human body [5]. The left ventricle is located below the left back of the right atrium, and the direction of its long axis slopes from the upper right to the lower left of the body with individual differences [6, 7]. Moreover, other organs and tissues around the heart also affected the observation field. Tracer uptakes were also observed in the right atrium, coronary sinus, and spine, which appeared as bright areas in the image [8, 9].

In order to facilitate clinical diagnosis and accurate quantitative analysis, the longitudinal image needs to be reoriented and rotated to obtain a cardiac short-axis (SA) image perpendicular to the left ventricular long axis [10]. Currently, steering studies focused on cardiac PET images are scarce, and most physicians adopted the traditional method manual for reorientation in clinical applications. SPECT as the mainstream imaging for cardiac disease, the related reorientation algorithms can be divided into traditional and automatic modes [11–17].

Traditional manual positioning mainly relies on the operator’s personal experience. According to visual judgment, the operator draws the long axis of the myocardium on horizontal long-axis (HLA) images and vertical long-axis (VLA) images to obtain the deviation angle, and then rotates the images to obtain SA images. However, this process is time-consuming, poorly repeatable, and error-prone [16].

The automatic algorithm obtains SA SPECT images through myocardial segmentation and model fitting. Employing techniques such as maximum radiation labeling or iterative clustering, the left ventricular myocardium could be segmented from SPECT images [11–13]. Subsequently, the left ventricular myocardium is fitted to an approximate model through model fitting, this step can calculate the left ventricular deviation angle, which is used to rotate the cardiac SPECT image and generate the SA SPECT image [14, 15].

Severe defects may lead to inaccurate positioning of the left ventricular long axis, so in clinical software such as Auto QUANT and Emory Cardiac Toolbox, the operator can manually adjust the long axis vector to ensure accurate reorientation [16–20]. Recently, the latest development of deep learning has also successfully introduced reorientations. For instance, Zhang et al. adopted convolutional neural networks (CNN) to predict six rigid body transformation parameters and obtained SA SPECT images by spatial transformation network [21].

However, the image quality of PET images is higher than that of SPECT images [22]. PET images can not only provide more detailed information about the left ventricle but also provide information about surrounding organs and tissues, which can serve as interference information and affect the accuracy of automatic reorientation [23–25]. Thus, the methods of SPECT may not be suitable for PET images.

Recent works related to myocardial PET images mostly focused on myocardial perfusion images (MPIs) and myocardial metabolic images (MMIs). In this work, MMI data was collected analyzed since it is widely used in China. The report of Su et al. has shown that segmenting the image into regions and extracting the heart part separately could improve the efficiency of subsequent segmentation [26]. Given that, we added the step of region division before traditional myocardial segmentation and model fitting.

In total, the purpose of our research is to find a set of automatic reorientation algorithms that are most suitable for 18 F-FDG PET MMIs. 6 methods of region division were adopted and compared, and the performances of 36 cross-combinations derived from 9 myocardial segmentation methods and 4 model fitting methods were evaluated.

## Methods and materials

### Study population

This study initially included 135 patients who underwent the 18 F-FDG PET/CT scanning on a Siemens Biography 16 PET/CT scanner at the Guangdong Provincial People’s Hospital from 2017 to 2019. However, 7 patients were excluded due to the following reasons: (1) poor image quality, or (2) incomplete clinical information. Finally, 128 PET/CT MMIs were included in the experiment, among them, 108 were coronary artery disease (CAD) patients, and the remaining 20 patients included heart failure, heart valvular disease, coronary artery aneurysm, and other cardiovascular diseases. Patient characteristics of 128 18 F-FDG PET MMIs are listed in Table 1. The study was approved by the institutional review board.

**Table 1:** Patient characteristics of 128 PET MMIs

Characteristic	*N* = 128
Age (years)	56.9 (5–81)
Gender	
Male	89.8% (115 / 128)
Female	10.2% (13 / 128)
Weight (kg)	63.5 (19.5–93)
Heart rate (times/minute)	75 (48–117)
Systolic blood pressure (mmHg)	115 (81–172)
Diastolic blood pressure (mmHg)	74 (47–109)
Hypertension	53 (41.4%)
Diabetes	35 (27.3%)
Smoking	43 (33.6%)
With CAD	108 (84.4%)
Without CAD	20 (15.6%)
MMIs without defects	48
MMIs with defects	49
MMIs with right ventricle excess uptake	31

CAD: Coronary artery disease;

MMI: Myocardial metabolism image

Two nuclear medicine doctors with 3 years of clinical experience utilized PMOD software to confirm the SA images corresponding to the PET MMIs data. The disputed cases were individually reviewed and reconfirmed by a nuclear medicine doctor with 13 years of clinical experience to obtain the reference reorientation images and reference reorientation angles for each case. The reference reorientation images were quantitatively analyzed by polar maps and the summed metabolism score (SMS). Cases with SMS > 2 were classified as patients with myocardial metabolic defect, and the cases with SMS < = 2 were divided into patients without myocardial metabolic defect and patients with right ventricle excess uptake after visual examination by two nuclear medicine doctors. The dataset was specifically divided into 48 patients without myocardial metabolic defect, 49 patients with myocardial metabolic defect, and 31 patients with right ventricle excess uptake.

Before the MMI scan, each patient underwent a glucose loading test. The patient’s blood glucose was measured after 45 min of the glucose loading test, and the insulin dosage by injecting insulin based on the patient’s blood glucose level. Check the patient’s blood sugar level every half hour, the 18 F-FDG was intravenously injected at a standard dose of 0.1mCi/Kg once the patient’s blood glucose level was at or below 7.8mmol/L. After a resting period of 90 min, each patient underwent a PET/CT scan. The scanning process was conducted sequentially, starting with a CT localization scan (120kVp, 10 mA), followed by a CT tomography scan (140kVp, 80 mA), and finally a 15-minute list mode PET scan. MMI images were reconstructed using attenuation-weighted ordered subset expectation maximization (two iterations, 24 subsets) and a Gaussian filter (FWHM = 5 mm). CT-based attenuation, scatter, decay, and random corrections were applied to the reconstructed images.

**Fig. 1:**
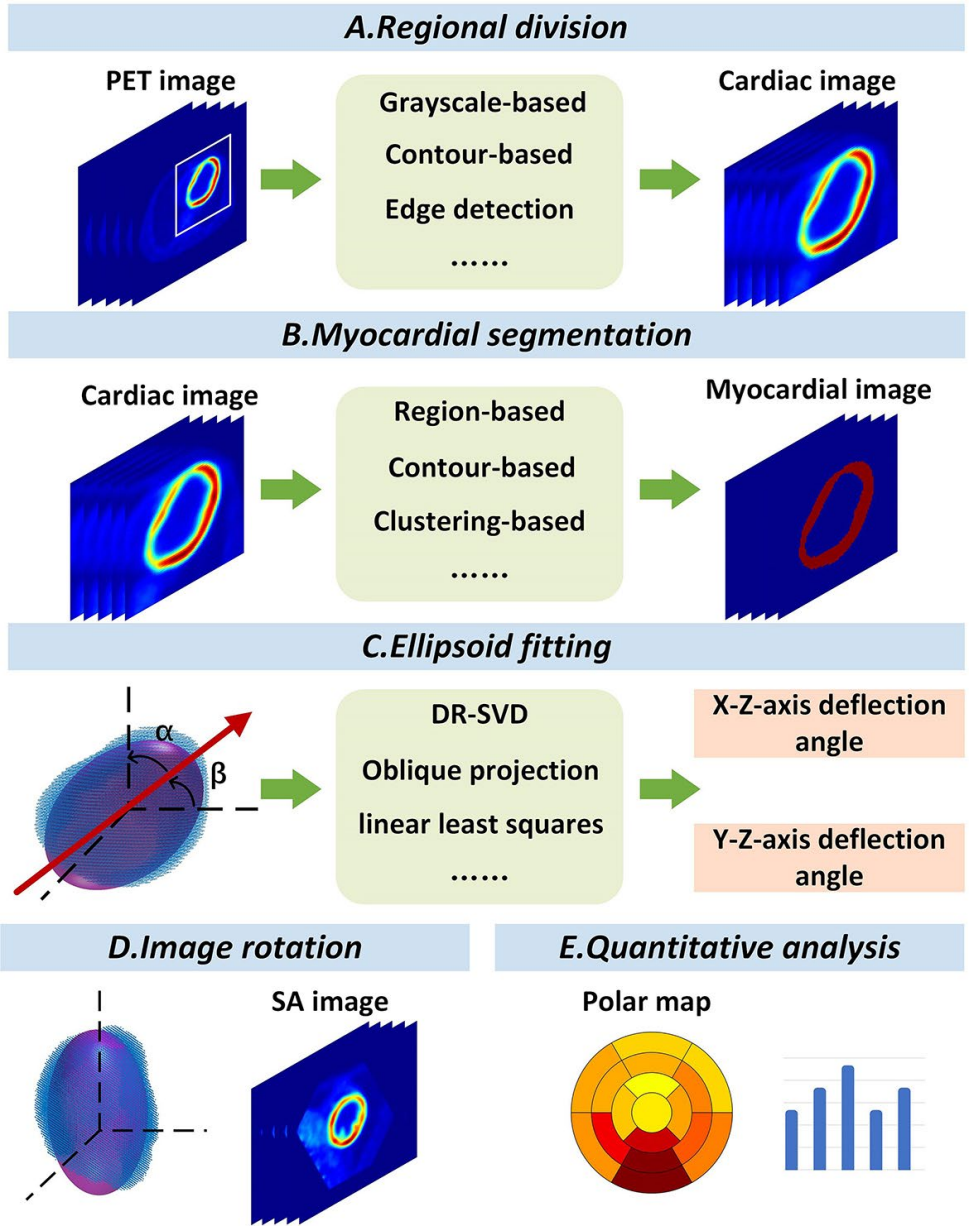
Overview of the cardiac PET image automatic redirection system, including regional division, myocardial segmentation, ellipsoid fitting, image rotation, and quantitative analysis

The proposed cardiac PET image automatic reorientation system consisted of five modules. As shown in Fig. 1, there are regional division, myocardial segmentation, ellipsoid fitting, image rotation and quantitative analysis. The inputs were 18 F-FDG PET MMIs, and the outputs were SA images and the quantitative analysis results.

### Regional division

The region-based division method is an image division technique that divides an image into several regions. Dividing the area containing the left ventricle in 18 F-FDG PET MMIs can remove the interference from other organs and tissues, which is beneficial for subsequent myocardial segmentation [26]. The myocardial PET images after regional division are resampled to a unified size of 80 * 80 * 64. One of the most common algorithms for region division is edge detection. We proposed the LVG-CED method, which improved the canny edge detection algorithm by introducing left ventricular position and size information as prior conditions. This method removed the areas that do not satisfy the conditions to improve partition efficiency and adopted the active contour algorithm with excellent performance in medical image division. Also, we compared some popular traditional algorithms for region partitioning of 18 F-FDG PET MMIs, including watershed [27], reconstruction closure (RC), iterative threshold segmentation (ITS) [28, 29], local fitting-based active contour models (LF-ACM) [30, 31], curve evolution (CE) [32], and LVG-CED.

### Myocardial segmentation

Through segmentation algorithms, we can segment the left ventricular myocardium from myocardial PET images. Segmentation algorithms are mainly divided into three categories: region-based methods, contour-based methods, and clustering-based methods [33, 34]. Region-based methods such as region partitioning and region growth are suitable for segmenting small regions. For the contour method, we selected an active contour driven by region scalable fitting and introduced a Gaussian energy Laplace operator optimization algorithm. Clustering-based algorithms are the most common and widely used segmentation methods. We compared the clustering performance of k-means and fuzzy C-means and used a particle swarm optimization algorithm to improve the speed and accuracy of clustering. In this study, a total of 9 segmentation algorithms were adopted for comparison, including maximum radioactivity sampling (MRS) [15], splitting and merging (SM) [35], region growth (RG) [34], multiplicative intrinsic component optimization (MICO) [36, 37], active contours driven by region-scalable fitting and optimized Laplacian of Gaussian energy (RSF-LOG) [38, 39], K-means, fuzzy C-means (FCM), particle swarm optimization K-means (PSO-K-means) [40], and particle swarm optimization fuzzy C-means (PSO-FCM) [41]. All the mentioned methods are fully automatic unsupervised segmentation algorithms that eliminate the need for manual adjustment or labeled data.

### Ellipsoid fitting

The shape of the human left ventricular myocardium is irregular, thus it is usually fitted by a hemisphere of a cylinder with a hemisphere, a cone, or an ellipsoid [11, 42, 43]. We chose to fit it as an ellipsoid, and the formula is shown in Eq. (1).

1 $${a_1}{x^2}+{a_2}{y^2}+{a_3}{z^2}+{a_4}xy+{a_5}xz+{a_6}yz+{a_7}x+{a_8}y+{a_9}z+{a_{10}}=0$$

Where$\:\:{\text{a}}_{\text{i}}$, 𝑖 = 1, 2, …10 are the 10 parameters of the ellipsoid. Based on the ellipsoid parameters, the long-axis deflection angle of the ellipsoid could be approximated as the left ventricular deflection angle, as shown in Eq. (2) and Eq. (3).

2 $$\alpha =\frac{1}{2}{\tan ^{ - 1}}\frac{{\sqrt 2 {a_6}}}{{{a_2} - {a_3}}}$$

3 $$\beta =\frac{1}{2}{\tan ^{ - 1}}\frac{{\sqrt 2 {a_4}}}{{{a_2} - {a_1}}}$$

Where 𝛼 is the X-Z axis deviation angle, and 𝛽 is the Y-Z axis deviation angle. The fitting algorithm of Douglas Rachford singular value decomposition (DR-SVD) [44], oblique projection (OP), linear least squares (LLS), and linear least squares singular value decomposition (LLS-SVD) were used separately.

### Image rotation

According to two deflection angles, we calculated the corresponding rotation matrices, and used affine transformation to rotate 18 F-FDG PET MMIs. The formulas as shown in Eqs. 4 and 5.

4 $${H_1}=\left[ {\begin{array}{*{20}{c}} 1&0&0 \\ 0&{\cos \alpha }&{ - \sin \alpha } \\ 0&{\sin \alpha }&{\cos \alpha } \end{array}} \right]$$

5 $${H_2}=\left[ {\begin{array}{*{20}{c}} {\cos \beta }&0&{\sin \beta } \\ 0&1&0 \\ { - \sin \beta }&0&{\cos \beta } \end{array}} \right]$$

where 𝛼 is the X-Z axis deviation angle, and 𝛽 is the Y-Z axis deviation angle. We cropped and resampled the rotated images to obtain SA images of the same size as 18 F-FDG PET MMIs.

### Quantitative analysis

Polar maps were used to quantitatively analyze the results of automatic orientation. Relied on the hemisphere plus cylinder model proposed by Carci et al. [42], the left ventricle apex was fitted by a hemisphere, the base and the mid-cavity was fitted by a cylinder. Referring to the criterions proposed by the American Heart Association [45], the polar map was divided into 17 segments and 3 blood supply vessel regions. The left anterior descending (LAD) consisted of 7 segments: 1, 2, 7, 8, 13, 14, 17. The right coronary artery (RCA) consisted of 5 segments: 3, 4, 9, 10, 15. The left circumflex (LCX) consisted of 5 segments: 5, 6, 11, 12, 16. The images manually positioned and rotated by clinical physicians were used as reference images. The similarity between the results obtained by our method and those obtained from reference images was calculated by Eq. (6).

6 $${\text{m}}=\left| {1 - \frac{{{p_r} - p{}_{o}}}{{{p_o}}}} \right| \times 100\%$$

where $\:{p}_{r}$ is the value of the polar map corresponding to the reference image, $\:{p}_{0}$ is the value of the polar map corresponding to the image obtained from our method, and $\:m$ is the similarity. We analyzed the results of 17 segments, 3 blood supply vessel regions, and the overall polar map in detail.

## Results

### Parameter-level analysis

**Fig. 2:**
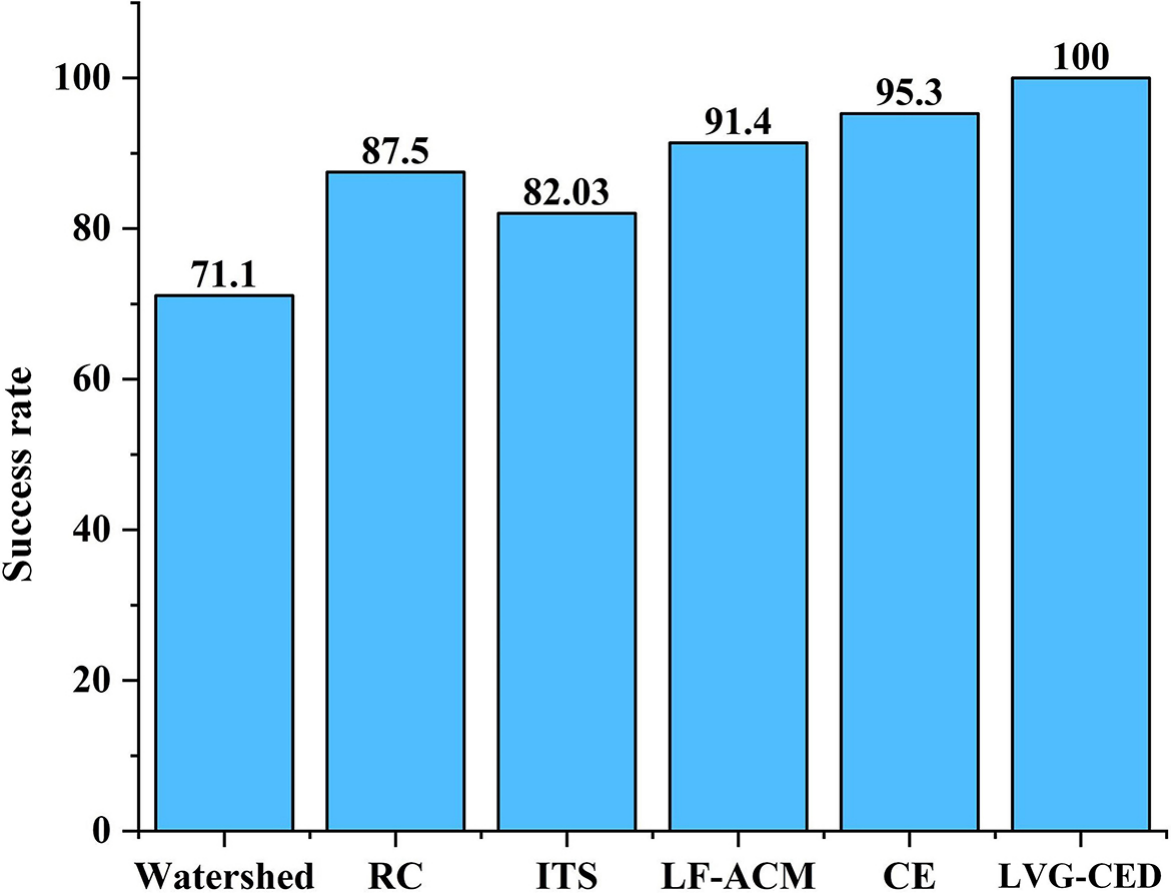
Statistical results of successfully dividing cardiac regions of 128 PET MMIs. RC: reconstruction closure, ITS: iterative threshold segmentation, LF-ACM: local fitting-based active contour models, CE: curve evolution, LVG-CED: left ventricular geometry-based canny edge detection

Figure 2 shows the statistical results of the regional division. The method was evaluated based on whether the entire heart region was completely divided. The success rate of LVG-CED was as high as 100%, and the success rate of CE was 95.3%.

**Fig. 3:**
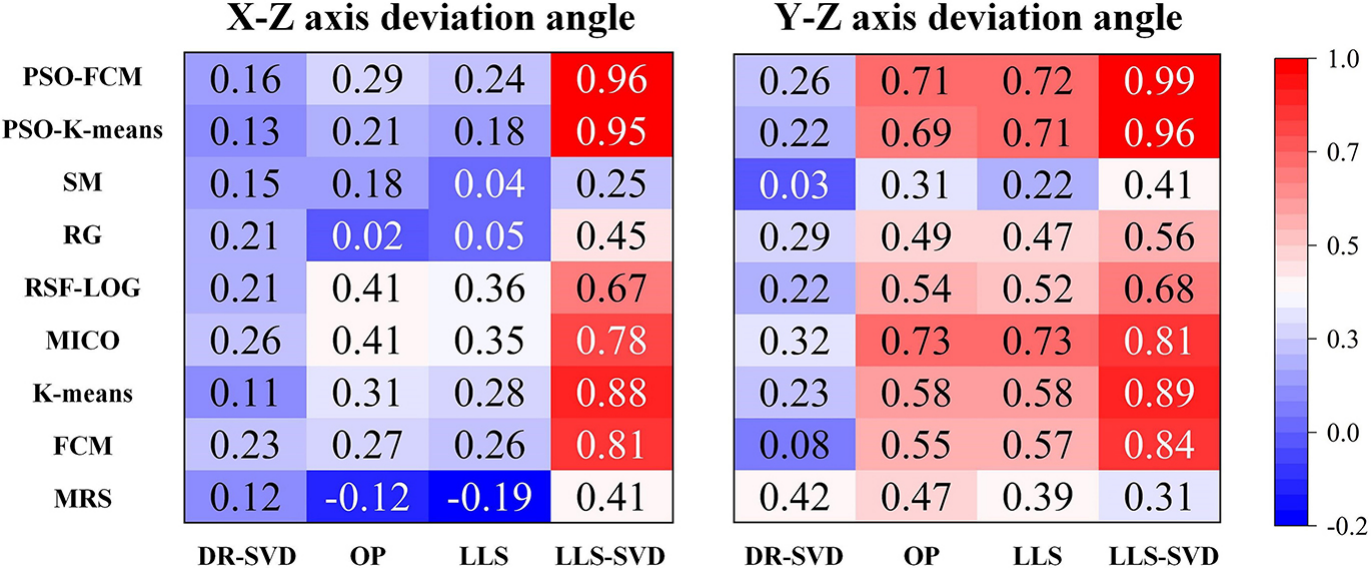
Heat map depicting the Pearson correlation coefficients of paired myocardial segmentation and ellipsoid fitting methods. MRS: maximum radioactivity sampling, SM: splitting and merging, RG region growth, MICO: multiplicative intrinsic component optimization, RSF-LOG: active contours driven by region-scalable fitting and optimized Laplacian of Gaussian energy, FCM: fuzzy C-means, PSO-K-means: particle swarm optimization K-means, PSO-FCM: particle swarm optimization fuzzy C-means, DR-SVD: Douglas Rachford singular value decomposition, OP: oblique projection, LLS: linear least squares, LLS-SVD: linear least squares singular value decomposition

The algorithm for the regional division was fixed as LVG-CED. We cross combined 9 myocardial segmentation methods and 4 ellipsoid fitting methods and compared the results with the manual method. The paired Pearson correlation coefficient (PCC) of different myocardial segmentation methods (in columns) and ellipsoid fitting methods (in rows) are shown in Fig. 3. Among them, the combination of PSO-FCM and LLS-SVD showed the highest performance (X-Z PCC: 0.96; Y-Z PCC: 0.99), followed by the combination of PSO-K-means and LLS-SVD (X-Z PCC: 0.95; Y-Z PCC: 0.96). The relevant results obtained by comparing cross combinations and the manual method are presented in Table 2, Supplementary Tables 1 and 2, which are PCC, Kendall correlation coefficient (KCC), and Spearman correlation coefficient (SCC), respectively. The results of similarity coefficient showed that the combination of PSO-FCM and LLS-SVD had an excellent agreement with the manual method (X-Z KCC: 0.87; Y-Z KCC: 0.95; X-Z SCC: 0.96; Y-Z SCC: 0.99).

**Table 2:** The Pearson correlation coefficients of cross-combined myocardial segmentation methods and ellipsoid fitting methods

Pearson	X-Z axis deviation angle				Y-Z axis deviation angle			
	DR-SVD	OP	LLS	LLS-SVD	DR-SVD	OP	LLS	LLS-SVD
MRS	0.12	-0.12	-0.20	0.40	0.42	0.47	0.39	0.31
SM	0.23	0.27	0.26	0.81	0.08	0.55	0.57	0.84
RG	0.11	0.31	0.28	0.88	0.23	0.58	0.58	0.89
MICO	0.26	0.41	0.35	0.78	0.32	0.73	0.73	0.80
RSF-LOG	0.21	0.40	0.36	0.67	0.20	0.54	0.52	0.68
K-means	0.21	0.02	0.05	0.45	0.29	0.49	0.47	0.56
FCM	0.15	0.18	0.04	0.25	0.03	0.30	0.22	0.40
PSO-K-means	0.13	0.20	0.18	0.95	0.22	0.71	0.70	0.96
PSO-FCM	0.16	0.29	0.24	**0.96**	0.26	0.70	0.70	**0.99**

MRS: Maximum radioactivity sampling, SM: Splitting and merging, RG: Region growth, MICO: Multiplicative intrinsic component optimization, RSF-LOG: Active contours driven by region-scalable fitting and optimized Laplacian of Gaussian energy, FCM: Fuzzy C-means, PSO-K-means: Particle swarm optimization K-means, PSO-FCM: Particle swarm optimization fuzzy C-means

**Fig. 4:**
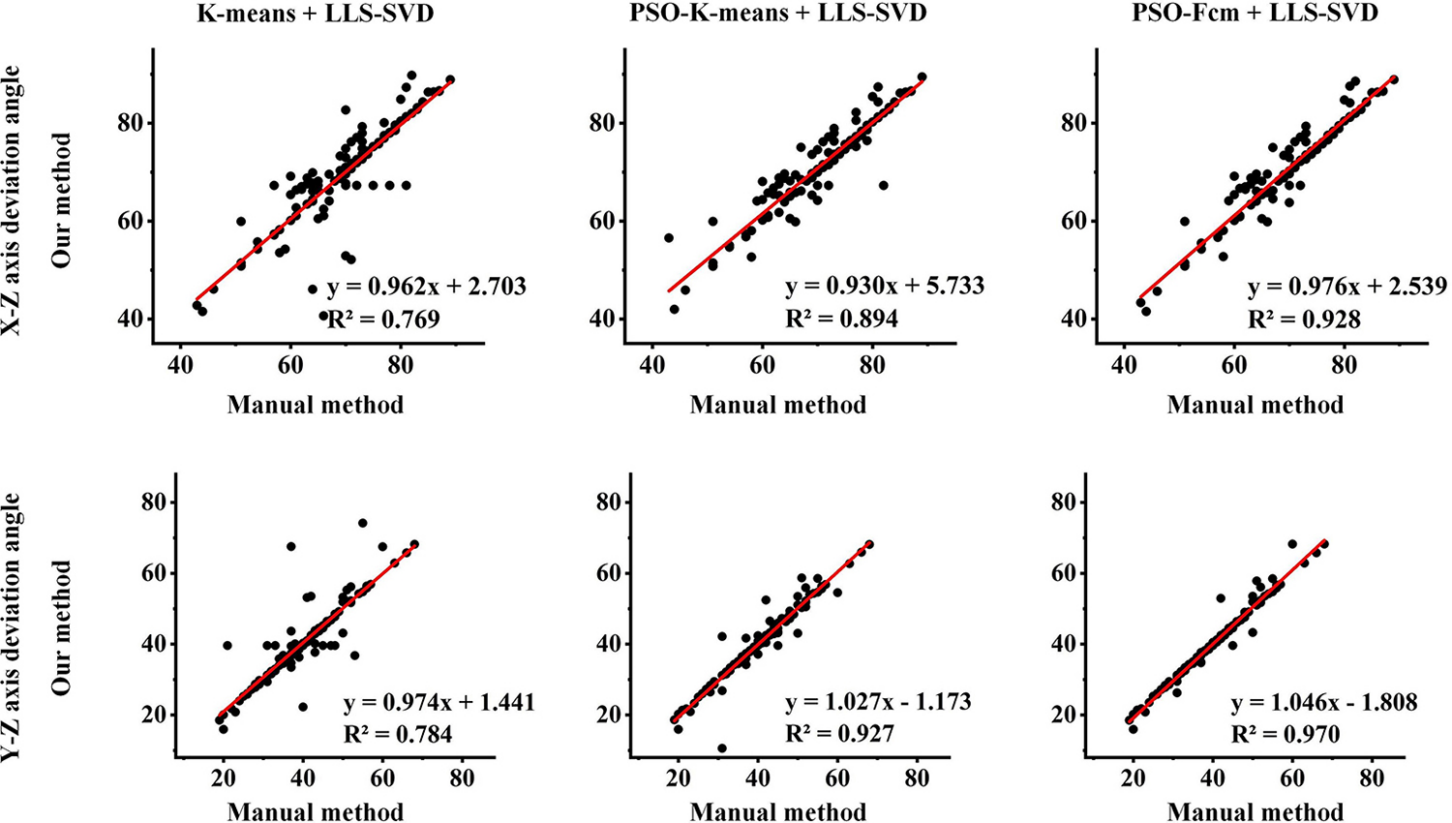
The linear regression plots between the predicted angles from our method and the reference angles from the manual method contain three best-performing combinations, in order: K-means + LLS-SVD, PSO-K-means + LLS-SVD, and PSO-FCM + LLS-SVD. The first row is the linear fitting results on the X-Z axis deflection angle, and the second row is the linear fitting results on the Y-Z axis deflection angle

To assess the accuracy and reliability of different combined traditional algorithms, Fig. 4 shows the linear fitting effects between the two deflection angles and the true value. The first column shows the linear regression coefficients of the predicted angle and the manual method on the X-Z deflection angle, and the second column shows the coefficients on the Y-Z deflection angle. The combinations from left to right were: K-means + LLS-SVD, PSO-K-means + LLS-SVD, and PSO-FCM + LLS-SVD. It is observed that the combination of PSO-FCM and LLS-SVD showed the best consistent results, with determination coefficients of 0.928 and 0.970, respectively.

Moreover, we compared our method with existing methods: topological goniometry (TG) [15] and MRS, the results of the correlation coefficients between the two reorientation angles are shown in Table 3. Our method performed best on all three correlation coefficients, while KCCs were 0.87 and 0.95, PCCs were 0.96 and 0.99, and SCCs were 0.96 and 0.99, respectively.

**Table 3:** The correlation coefficient between existing methods and our method

	X-Z axis deviation angle			Y-Z axis deviation angle		
	Kendall	Pearson	Spearman	Kendall	Pearson	Spearman
TG	0.34	0.39	0.47	0.38	0.40	0.51
MRS	0.24	0.40	0.34	0.25	0.31	0.33
**Our Method**	**0.87**	**0.96**	**0.96**	**0.95**	**0.99**	**0.99**

TG: Topological goniometry

MRS: Maximum radioactivity sampling

### Clinical quantitative analysis

To further verify the reliability of our method, Table 4 shows the similarity between the selected optimal combination (LVG-CED, PSO-FCM, and LLS-SVD) and the manual method for quantitative analysis of the poplar map. Similarities of all 17 segments were more than 96%, the similarity of the mid inferoseptal had the highest similarity (98.89%) among 17 segments, RCA had the highest similarity (98.18%) among 3 blood supply vessel regions, and the overall average similarity of the left ventricle was 97.84%.

**Table 4:** The similarities of the 17 segments and the 3 blood supply vessel regions among 128 patients

Segment	1	2		3	4		5
Similarity (%)	96.74 ± 7.20	98.01 ± 5.25		98.64 ± 3.58	97.16 ± 10.19		96.89 ± 10.91
Segment	6	7		8	9		10
Similarity (%)	97.72 ± 4.61	97.47 ± 7.08		97.95 ± 6.26	**98.89 ± 3.82**		97.75 ± 4.89
Segment	11	12		13	14		15
Similarity (%)	97.59 ± 7.31	98.50 ± 3.13		96.84 ± 17.29	98.55 ± 5.96		98.47 ± 6.49
Segment	16	17					
Similarity (%)	97.82 ± 9.46	98.27 ± 5.13					
blood supply vessel region	LAD		RCA			LCX	
Similarity (%)	97.69 ± 6.16		**98.18 ± 4.54**			97.70 ± 5.51	
Total Similarity (%)	**97.84 ± 4.86**						

LAD: left anterior descending

RCA: right coronary artery

LCX: left circumflex

**Fig. 5:**
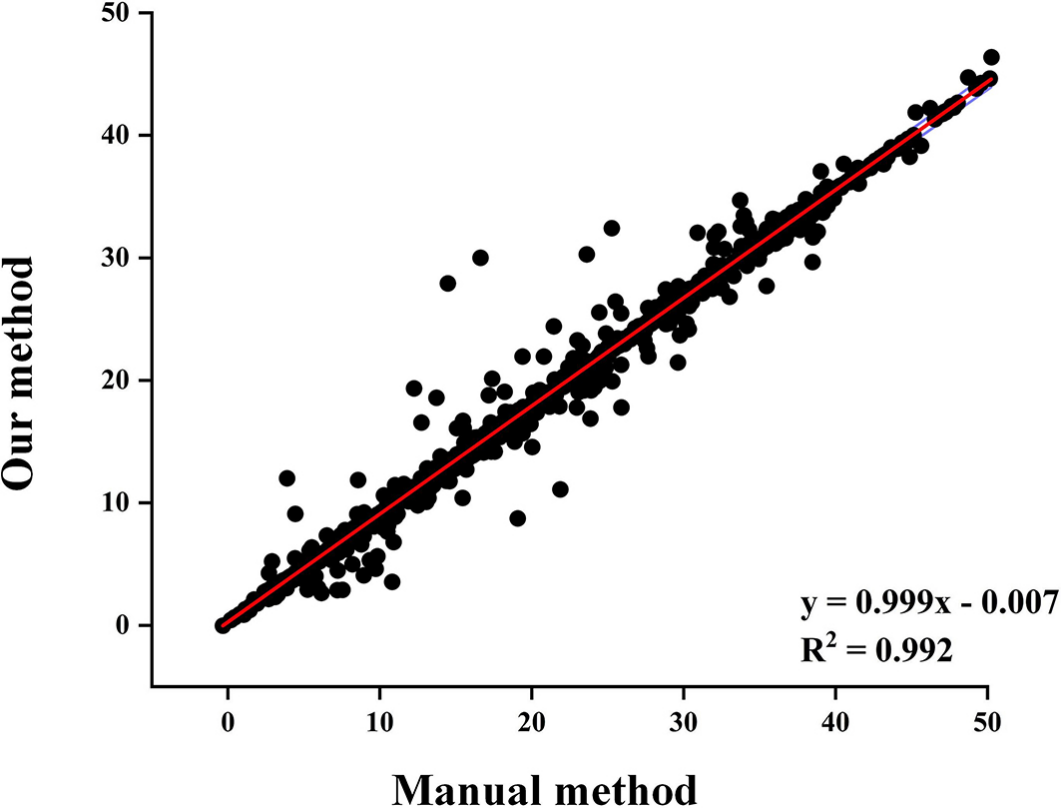
The linear regression plot between the 2176 segment values calculated using our method and the reference segment values using the manual method

Figure 5 shows the linear regression result of 17 segments of 128 patients’ polar maps (2142 segments in total). The optimal combination (LVG-CED, PSO-FCM, and LLS-SVD) was highly consistent with the manual method, and the determination coefficient was as high as 0.992.

### Image-level analysis

**Fig. 6:**
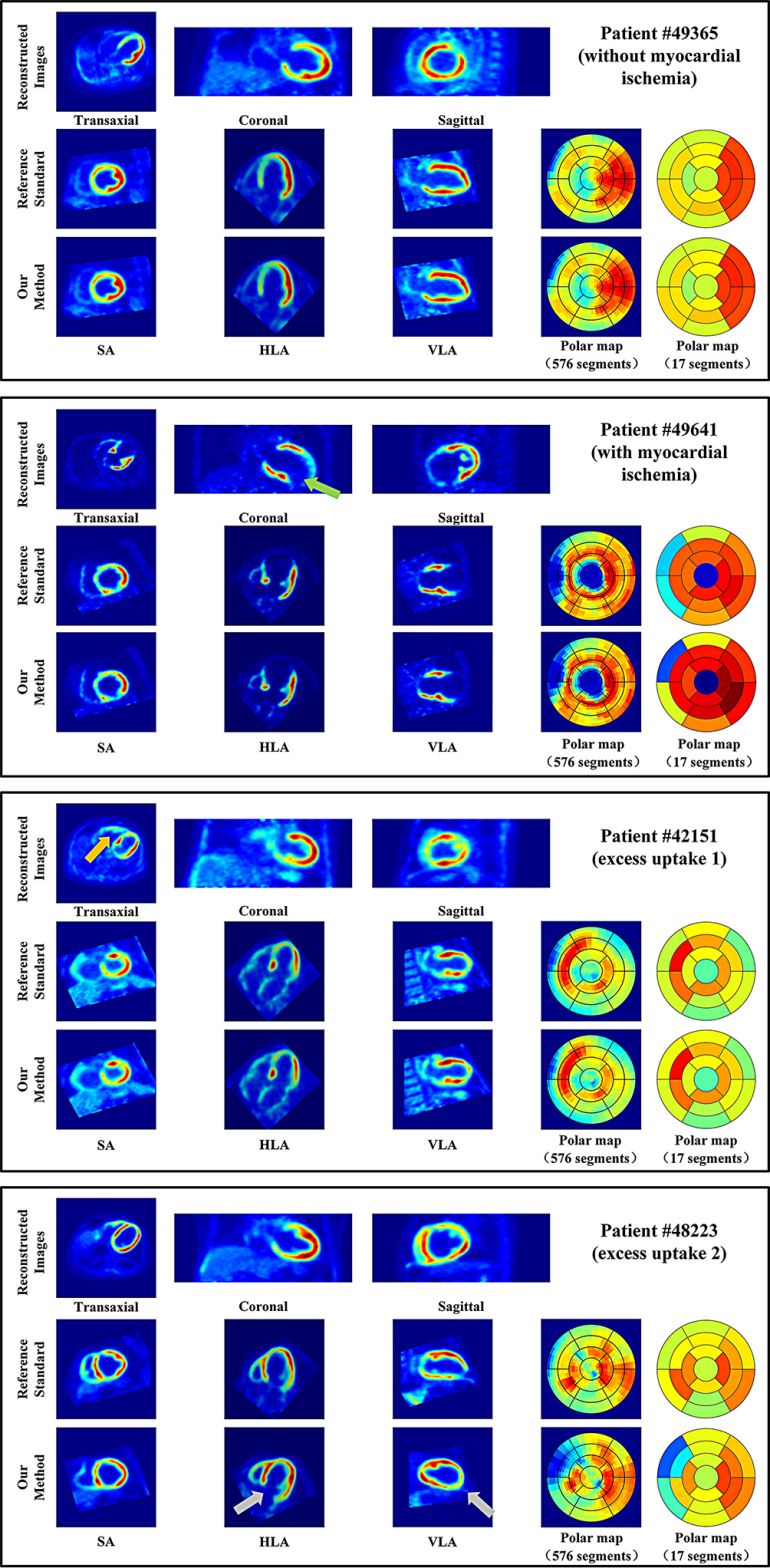
Reconstructed images, reference reoriented images by the manual method, and reoriented images by our method for four PET MMIs: patient #49,365 without defects, patient #49,641 with defects, while patient #42,151 and patient #48,223 have excess uptake. For each, (i) reconstructed images are shown (From left to right): Transaxial, coronal, and sagittal 3 orthogonal orientations, (ii) reference reoriented images by the manual method and (iii) reoriented images by our method are shown (From left to right): SA, HLA, VLA, polar map (576 segments) and polar map (17 segments). SA: short axis, HLA: horizontal long axis, VLA: vertical long axis

Figure 6 shows the PET images of four patients, including reconstructed images, reference images, images obtained by our method (LVG-CED, PSO-FCM, and LLS-SVD), and corresponding polar maps of SA images.

Patient #49,365 without defects, mild ischemia of the basal anterolateral didn’t affect the automatic reorientation, and the images obtained by our method were highly consistent with reference images. Patient #49,641 and patient #42,151 are two cases with interferences. The green shear in Fig. 6 pointed out an apical ischemia, and the yellow shear in Fig. 6 pointed out an excessive uptake of the right ventricle. Both cases had interferences with the automatic reorientation, but our method still exhibited good performance. However, when the concentration of excess uptakes and left ventricular uptakes was similar (patient #48,223), it might still affect the effectiveness of the automatic redirection and cause inaccuracies in subsequent quantitative analysis.

### Specific classification analysis

Table 5 shows the correlation analysis results obtained by comparing our method and the manual method under three different image classifications. It included MMIs without defects, MMIs with defects, and MMIs with right ventricle excess uptake. our method achieved high consistency with the manual method in all three types of patients. The similarity of MMIs without defects was the highest, and the similarity of MMIs with right ventricle excess uptake was the lowest, while KCCs were 0.736 and 0.891, PCCs were 0.948 and 0.968, and SCCs were 0.879 and 0.964, respectively.

**Table 5:** The 3 correlation coefficients under 3 types of image classification

	X-Z axis deviation angle			Y-Z axis deviation angle		
	Kendall	Pearson	Spearman	Kendall	Pearson	Spearman
MMIs without defects	**0.919**	**0.984**	**0.980**	**0.986**	**0.999**	**0.998**
MMIs with defects	0.859	0.959	0.959	0.932	0.976	0.982
MMIs with right ventricle excess uptake	0.736	0.948	0.879	0.891	0.968	0.964

MMI: Myocardial metabolic image

## Discussion

In this study, we developed an algorithm system for automatic left ventricular reorientation and simultaneous quantitative analysis on 18 F-FDG PET MMIs. Our algorithm system included five parts: regional division, myocardial segmentation, ellipsoid fitting, image rotation, and quantitative analysis. We compared 6 regional segmentation methods and 36 cross combinations (9 myocardial segmentation methods and 4 ellipsoid fitting methods) and ultimately chose the best performing method as our method: LVG-CED, PSO-FCM, and LLS-SVD. Compared to the manual method, our method showed a high consistency at both the image level and the quantitative analysis level.

In the regional division module, we chose LVG-CED in our system. The prior knowledge of heart contour can improve the accuracy of detection [46]. In our study, the prior knowledge provided the information of left ventricular location and size, and edge detection effectively detected the left ventricular myocardial edge. The success rate of partitioning the left ventricular region on our dataset was 100%, which was higher than all other traditional partitioning algorithms (Fig. 2).

In previous studies, various algorithms such as maximum radiation sampling, clustering, feature extraction, threshold partitioning, etc. have been used for myocardial segmentation [10–20]. However, due to different datasets, different preprocessing methods, and other variables, it is not possible to integrate and compare these methods. In our study, we used a total of 9 popular traditional segmentation algorithms for myocardial segmentation. In terms of model fitting selection, a cylinder with a hemisphere, a cone, or a ellipsoid have been used to fit the model of left ventricular myocardium, but the fitting effect of the first two models was poor in the areas with severe ischemia, which affects the accuracy of subsequent reorientation [11, 42]. The ellipsoid was similar in shape to the left ventricle and less affected by defects [43], and it was selected as the myocardium fitting model. The combination of myocardial segmentation and ellipsoid fitting had the best similarity coefficient among PSO-FCM and LLS-SVD. On the X-Z axis deviation angle, KCC, PCC and SCC were 0.87, 0.96 and 0.96 respectively, and were 0.95, 0.99 and 0.99 on the Y-Z axis deviation angle as shown in Table 2, Supplementary Tables 1 and 2.

At present, software for fully automatic PET cardiac image reorientation has not been applied in clinical diagnosis. Through semi-automatic methods, doctors manually adjust the results of the software’s automatic segmentation of fixed points to determine rotation parameters and obtain SA images, it has low repeatability and large errors [16]. However, due to the difficulty in obtaining PET data, there are rare studies focused on automatic reorientation of 18 F-FDG PET MMIs, and existing studies mainly focus on SPECT images. For instance, Zhang et al. used 322 SPECT images, and the determination coefficients of three rotation parameters were 0.928, 0.958, and 0.994 respectively [21]. Zhu et al. used 254 SPECT images, and the determination coefficients of three rotation parameters were 0.987, 0.990, and 0.996 respectively [22]. On our data set of 128 cases of 18 F-FDG PET MMIs, our method achieved similar results with the determination coefficients of 0.928 and 0.970 (Fig. 4).

The polar map, as the best semi quantitative method for visual interpretation of regional left ventricular abnormalities, was selected as the clinical quantitative analysis method in our study [47]. The linear fitting results of Fig. 5 indicated that the polar map values obtained through our method were highly consistent with the results obtained through the manual method (determination coefficient: 0.9915). The similarities were higher than 96% in all 17 segments, with the mid inferoseptal segment having the highest similarity (98.89%). Besides, we found that the similarity of RCA was the highest among the 3 blood supply vessel regions, at 98.18%, the total similarity of the polar maps corresponding to 128 18 F-FDG PET MMIs was 97.84% (Table 4).

In the study of automatic reorientation of the left ventricle, there are two main difficulties: (1) Defects, which may lead to insufficient myocardial imaging on PET images, increasing the difficulty of model fitting and introducing errors in ellipsoid fitting [11, 15]; (2) Excess uptakes, when additional tracer uptake is present in the organs and tissues surrounding the left ventricle, may be mistaken for the left ventricle, introducing errors in myocardial segmentation. Either of these situations can result in the failure of automatic redirection [16–18]. Based on these two difficulties, we divide images into three categories: MMIs without defects, MMIs with defects and MMIs with right ventricle excess uptake. As shown in Fig. 6, our method performed well under all 3 classifications, and the SA images and polar maps obtained by our method had good agreement with those obtained by the manual method. Also notice that MMIs with defects showed higher correlation coefficients than that from MMIs with right vehicle excess uptake (KCC: 0.859 and 0.932 vs. 0.736 and 0.891, PCC: 0.959 and 0.976 vs. 0.948 and 0.968, SCC: 0.959 and 0.982 vs. 0.879 and 0.964). The errors caused by defects can be reduced to some extent by ellipsoid fitting, but the excess uptake is not easy to distinguish from the left ventricular myocardium, and the impact of the results is greater.

Our study had several limitations. First, as shown in Fig. 6, when the distances between the right ventricle and the left ventricle were close and the uptake intensities were similar, myocardial segmentation errors may occur, leading to errors in subsequent automatic reorientation. However, due to the close distance, the angle errors were still less than 20 degrees. Second, the methods we used are all traditional algorithms. In recent years, deep learning has shown excellent performance in medical image processing. In subsequent experiments, we hope to collect more PET data and introduce deep learning into automatic reorientation to further compare and organize with traditional algorithms.

## Conclusion

We developed an algorithm system for automatic reorientation and quantitative analysis of 18 F-FDG PET MMIs. Our results showed that the system was highly consistent with the manual method at both the image level and the quantitative analysis level. Further, it achieved good performance on three different image classifications (MMIs without defects, MMIs with defects, and MMIs with right ventricle excess uptake).

### Electronic supplementary material

Below is the link to the electronic supplementary material.

Supplementary Material 1

## Data Availability

The datasets utilized or analyzed in the present study are accessible from the corresponding author upon a reasonable request.

## Abbreviations

PET: Positron emission tomography
SPECT: Single-photon emission computed tomography
SA: Short-axis
HLA: Horizontal long-axis
VLA: Vertical long-axis
MMI: Myocardial metabolic image
MPI: Myocardial perfusion images
CAD: Coronary artery disease
SMS: Summed metabolism score
PCC: Pearson correlation coefficient
KCC: Kendall correlation coefficient
SCC: Spearman correlation coefficient
