# Supplementary material for: Automatic reorientation to generate short-axis myocardial PET images
Source: EJNMMI Phys. 2024 Aug 2;11:70. doi: 10.1186/s40658-024-00673-9 (PMC11294504; doi:10.1186/s40658-024-00673-9)
Supplement: Supplementary file 1 — Supplementary Material 1 [file 40658_2024_673_MOESM1_ESM.docx]

**Supplementary Table 1**. The Kendall correlation coefficients of cross-combined myocardial segmentation methods and ellipsoid fitting methods

| **Kendall** | X-Z axis deviation angle | | | | Y-Z axis deviation angle | | | |
| --- | --- | --- | --- | --- | --- | --- | --- | --- |
|  | DR-SVD | OP | LLS | LLS-SVD | DR-SVD | OP | LLS | LLS-SVD |
| MRS | 0.09 | -0.12 | -0.17 | 0.24 | 0.33 | 0.37 | 0.33 | 0.25 |
| SM | 0.01 | -0.04 | -0.04 | 0.68 | 0.08 | 0.47 | 0.48 | 0.77 |
| RG | -0.05 | -0.02 | -0.04 | 0.77 | 0.17 | 0.48 | 0.49 | 0.81 |
| MICO | 0.08 | 0.27 | 0.20 | 0.65 | 0.19 | 0.59 | 0.59 | 0.72 |
| RSF-LOG | 0.10 | 0.11 | 0.10 | 0.44 | 0.10 | 0.39 | 0.37 | 0.53 |
| K-means | 0.10 | 0.02 | 0.04 | 0.33 | 0.21 | 0.37 | 0.36 | 0.45 |
| FCM | 0.07 | 0.06 | -0.03 | 0.25 | 0.03 | 0.24 | 0.22 | 0.38 |
| PSO-K-means | -0.04 | -0.08 | -0.10 | 0.84 | 0.15 | 0.56 | 0.56 | 0.91 |
| PSO-FCM | -0.04 | -0.05 | -0.10 | **0.87** | 0.18 | 0.55 | 0.55 | **0.95** |

MRS: maximum radioactivity sampling, SM: splitting and merging, RG: region growth, MICO: multiplicative intrinsic component optimization, RSF-LOG: active contours driven by region-scalable fitting and optimized Laplacian of Gaussian energy, FCM: fuzzy C-means, PSO-K-means: particle swarm optimization K-means, PSO-FCM: particle swarm optimization fuzzy C-means

**Supplementary Table 2**. The Spearman correlation coefficients of cross-combined myocardial segmentation methods and ellipsoid fitting methods

| **Spearman** | X-Z axis deviation angle | | | | Y-Z axis deviation angle | | | |
| --- | --- | --- | --- | --- | --- | --- | --- | --- |
|  | DR-SVD | OP | LLS | LLS-SVD | DR-SVD | OP | LLS | LLS-SVD |
| MRS | 0.10 | -0.17 | -0.21 | 0.34 | 0.44 | 0.51 | 0.46 | 0.33 |
| SM | 0.01 | -0.05 | -0.05 | 0.81 | 0.10 | 0.60 | 0.61 | 0.88 |
| RG | -0.07 | -0.02 | -0.05 | 0.89 | 0.22 | 0.62 | 0.62 | 0.90 |
| MICO | 0.10 | 0.34 | 0.25 | 0.80 | 0.27 | 0.74 | 0.74 | 0.86 |
| RSF-LOG | 0.14 | 0.15 | 0.13 | 0.60 | 0.14 | 0.53 | 0.51 | 0.70 |
| K-means | 0.14 | 0.03 | 0.05 | 0.46 | 0.30 | 0.53 | 0.51 | 0.57 |
| FCM | 0.10 | 0.08 | -0.04 | 0.34 | 0.03 | 0.34 | 0.30 | 0.51 |
| PSO-K-means | -0.05 | -0.10 | -0.12 | 0.94 | 0.20 | 0.71 | 0.71 | 0.97 |
| PSO-FCM | -0.05 | -0.06 | -0.12 | **0.96** | 0.24 | 0.69 | 0.70 | **0.99** |

MRS: maximum radioactivity sampling, SM: splitting and merging, RG: region growth, MICO: multiplicative intrinsic component optimization, RSF-LOG: active contours driven by region-scalable fitting and optimized Laplacian of Gaussian energy, FCM: fuzzy C-means, PSO-K-means: particle swarm optimization K-means, PSO-FCM: particle swarm optimization fuzzy C-means
